# Supplementary material for: De novo design of a macrocycle-induced dimerization system for cellular control
Source: Nat Commun. 2026 May 18;17:6683. doi: 10.1038/s41467-026-71345-8 (PMC13385886; doi:10.1038/s41467-026-71345-8)
Supplement: Supplementary file 2 — Description of Additional Supplementary Information [file 41467_2026_71345_MOESM2_ESM.pdf]

Description of Additional Supplementary Information

Title: Supplementary Data 1
Description: Sequence information for plasmids used in transcriptional assay in Figure 4.
