# Supplementary material for: De novo design of a macrocycle-induced dimerization system for cellular control
Source: Nat Commun. 2026 May 18;17:6683. doi: 10.1038/s41467-026-71345-8 (PMC13385886; doi:10.1038/s41467-026-71345-8)
Supplement: Supplementary file 4 — Reporting Summary [file 41467_2026_71345_MOESM4_ESM.pdf]

nature portfolio

Corresponding author(s): David Baker

Last updated by author(s): 3/19/2026

Reporting Summary

Nature Portfolio wishes to improve the reproducibility of the work that we publish. This form provides structure for consistency and transparency in reporting. For further information on Nature Portfolio policies, see our Editorial Policies and the Editorial Policy Checklist.

Statistics

For all statistical analyses, confirm that the following items are present in the figure legend, table legend, main text, or Methods section.

n/a	Confirmed	
☐	☒	The exact sample size (n) for each experimental group/condition, given as a discrete number and unit of measurement
☐	☒	A statement on whether measurements were taken from distinct samples or whether the same sample was measured repeatedly
☒	☐	The statistical test(s) used AND whether they are one- or two-sided<br>*Only common tests should be described solely by name; describe more complex techniques in the Methods section.*
☒	☐	A description of all covariates tested
☒	☐	A description of any assumptions or corrections, such as tests of normality and adjustment for multiple comparisons
☐	☒	A full description of the statistical parameters including central tendency (e.g. means) or other basic estimates (e.g. regression coefficient) AND variation (e.g. standard deviation) or associated estimates of uncertainty (e.g. confidence intervals)
☒	☐	For null hypothesis testing, the test statistic (e.g. F, t, r) with confidence intervals, effect sizes, degrees of freedom and P value noted<br>*Give P values as exact values whenever suitable.*
☒	☐	For Bayesian analysis, information on the choice of priors and Markov chain Monte Carlo settings
☒	☐	For hierarchical and complex designs, identification of the appropriate level for tests and full reporting of outcomes
☒	☐	Estimates of effect sizes (e.g. Cohen's d, Pearson's r), indicating how they were calculated

Our web collection on statistics for biologists contains articles on many of the points above.

Software and code

Policy information about availability of computer code

Data collection	ITC data was collected on an automated Microcal PEAQ-ITC v.1.4.0. Luminescence data was collected on a Synergy Neo2 plate reader with BioTek Gen5 software version 3.14. Refeyn AcquireMP 9.1.0.0 for mass photometry data. Code made for macrocyclic peptide and protein design is available for download at https://files.ipd.uw.edu/pub/macrocycle_cid/code.zip.
Data analysis	All structural images for figures were generated with The PyMOL Molecular Graphics System, Version 2.5.8, Schrödinger, LLC. Data were analyzed, plotted, and processed with python (3.9.6), matplotlib (3.7.1), seaborn (0.12.2), and pandas (2.0.2). ITC data curves were fit with Microcal PEAQ-ITC v1.4.0. Bioconfirm 10.0 and Skyline 25.1.0.237 were used for mass spec data analysis. DiscoverMP v2025 R1 was used for analysis of mass photometry data. Flow cytometry data were analyzed with FlowJo [v10.9.0]. GraphPad prism (v10.5.0) was used for plotting and curve fitting for cell assay data. Crystallographic data was processed with Coot (0.9.6), Phaser and Phenix (1.20).

For manuscripts utilizing custom algorithms or software that are central to the research but not yet described in published literature, software must be made available to editors and reviewers. We strongly encourage code deposition in a community repository (e.g. GitHub). See the Nature Portfolio guidelines for submitting code & software for further information.

Data

Policy information about availability of data

All manuscripts must include a data availability statement. This statement should provide the following information, where applicable:

- Accession codes, unique identifiers, or web links for publicly available datasets
- A description of any restrictions on data availability
- For clinical datasets or third party data, please ensure that the statement adheres to our policy

Source data are provided with this paper, including all experimental data presented in this manuscript. The protein crystallographic data generated in this study have been deposited in the PDB under accession codes 8VX7 (https://doi.org/10.2210/pdb8VX7/pdb), 8TM9 (https://doi.org/10.2210/pdb8TM9/pdb), and 8TLP (https://doi.org/10.2210/pdb8TLP/pdb). The macrocycle crystallographic data generated in this study have been deposited in the Crystallographic Data Centre (CCDC) with deposition numbers 2354604 [https://www.ccdc.cam.ac.uk/structures/Search?ccdc=2354604], 2354605 [https://www.ccdc.cam.ac.uk/structures/Search?ccdc=2354605], 2354606 [https://www.ccdc.cam.ac.uk/structures/Search?ccdc=2354606], 2354607 [https://www.ccdc.cam.ac.uk/structures/Search?ccdc=2354607], 2354608 (MC4, [https://www.ccdc.cam.ac.uk/structures/Search?ccdc=2354608], 2354609 [https://www.ccdc.cam.ac.uk/structures/Search?ccdc=2354609], 2360904 [https://www.ccdc.cam.ac.uk/structures/Search?ccdc=2360904], and 2354610 [https://www.ccdc.cam.ac.uk/structures/Search?ccdc=2354610].

Research involving human participants, their data, or biological material

Policy information about studies with human participants or human data. See also policy information about sex, gender (identity/presentation), and sexual orientation and race, ethnicity and racism.

Reporting on sex and gender	*Use the terms sex (biological attribute) and gender (shaped by social and cultural circumstances) carefully in order to avoid confusing both terms. Indicate if findings apply to only one sex or gender; describe whether sex and gender were considered in study design; whether sex and/or gender was determined based on self-reporting or assigned and methods used.* *Provide in the source data disaggregated sex and gender data, where this information has been collected, and if consent has been obtained for sharing of individual-level data; provide overall numbers in this Reporting Summary. Please state if this information has not been collected.* *Report sex- and gender-based analyses where performed, justify reasons for lack of sex- and gender-based analysis.*
Reporting on race, ethnicity, or other socially relevant groupings	*Please specify the socially constructed or socially relevant categorization variable(s) used in your manuscript and explain why they were used. Please note that such variables should not be used as proxies for other socially constructed/relevant variables (for example, race or ethnicity should not be used as a proxy for socioeconomic status).* *Provide clear definitions of the relevant terms used, how they were provided (by the participants/respondents, the researchers, or third parties), and the method(s) used to classify people into the different categories (e.g. self-report, census or administrative data, social media data, etc.)* *Please provide details about how you controlled for confounding variables in your analyses.*
Population characteristics	*Describe the covariate-relevant population characteristics of the human research participants (e.g. age, genotypic information, past and current diagnosis and treatment categories). If you filled out the behavioural & social sciences study design questions and have nothing to add here, write "See above."*
Recruitment	*Describe how participants were recruited. Outline any potential self-selection bias or other biases that may be present and how these are likely to impact results.*
Ethics oversight	*Identify the organization(s) that approved the study protocol.*

Note that full information on the approval of the study protocol must also be provided in the manuscript.

Field-specific reporting

Please select the one below that is the best fit for your research. If you are not sure, read the appropriate sections before making your selection.

☒ Life sciences ☐ Behavioural & social sciences ☐ Ecological, evolutionary & environmental sciences

For a reference copy of the document with all sections, see nature.com/documents/nr-reporting-summary-flat.pdf

Life sciences study design

All studies must disclose on these points even when the disclosure is negative.

Sample size	Samples sizes are listed in figure captions. No statistical methods were used to pre-determine sample size.
Data exclusions	No data were excluded.
Replication	Key experiments were replicated with at least 2 independent runs.
Randomization	No randomization was necessary for the experiments in this study.

Blinding	Blinding was not relevant for the experiments in this study.

Reporting for specific materials, systems and methods

We require information from authors about some types of materials, experimental systems and methods used in many studies. Here, indicate whether each material, system or method listed is relevant to your study. If you are not sure if a list item applies to your research, read the appropriate section before selecting a response.

Materials & experimental systems

n/a	Involved in the study
x	Antibodies
	x Eukaryotic cell lines
x	Palaeontology and archaeology
x	Animals and other organisms
x	Clinical data
x	Dual use research of concern
x	Plants

Methods

n/a	Involved in the study
x	ChIP-seq
	x Flow cytometry
x	MRI-based neuroimaging

Eukaryotic cell lines

Policy information about cell lines and Sex and Gender in Research

Cell line source(s)	HEK293T (ATCC, CRL-3216)
Authentication	HEK293T cells were authenticated by the manufacturer by STR profiling.
Mycoplasma contamination	HEK293T cells were tested for mycoplasm contamination by the manufacturer and was not detected.
Commonly misidentified lines (See ICLAC register)	No commonly misidentified cell lines were used in this study.

Plants

Seed stocks	*Report on the source of all seed stocks or other plant material used. If applicable, state the seed stock centre and catalogue number. If plant specimens were collected from the field, describe the collection location, date and sampling procedures.*
Novel plant genotypes	*Describe the methods by which all novel plant genotypes were produced. This includes those generated by transgenic approaches, gene editing, chemical/radiation-based mutagenesis and hybridization. For transgenic lines, describe the transformation method, the number of independent lines analyzed and the generation upon which experiments were performed. For gene-edited lines, describe the editor used, the endogenous sequence targeted for editing, the targeting guide RNA sequence (if applicable) and how the editor was applied.*
Authentication	*Describe any authentication procedures for each seed stock used or novel genotype generated. Describe any experiments used to assess the effect of a mutation and, where applicable, how potential secondary effects (e.g. second site T-DNA insertions, mosiacism, off-target gene editing) were examined.*

Flow Cytometry

Plots

Confirm that:

- [x] The axis labels state the marker and fluorochrome used (e.g. CD4-FITC).
- [x] The axis scales are clearly visible. Include numbers along axes only for bottom left plot of group (a 'group' is an analysis of identical markers).
- [x] All plots are contour plots with outliers or pseudocolor plots.
- [x] A numerical value for number of cells or percentage (with statistics) is provided.

Methodology

Sample preparation	HEK293T cells (ATCC) were cultured in tissue culture-treated 384-well plates. Each well contained 50 µL of HEK293T cells at a density of 600,000 cells/mL in cell media (Dulbecco's Modified Eagle Medium with Glutamax (DMEM, Thermo Fisher), 10% fetal bovine serum, heat inactivated (FBS, Thermo Fisher), 1X penicillin strep media (10,000 U/ml, Thermo Fisher)). At approximately 80% confluency cells were then transfected with the respective vectors using either polyethylenimine (PEI) or Viafect at a 3:1 ratio (transfection reagent to DNA). After approximately 10 minutes the plasmid transfection reagent was added to cultured cells. Cells were incubated with plasmid and transfection reagent for approximately 24 h at 37℃. After

incubation with the transfection reagent, media was removed and replaced with new cell media. MC1 was serially diluted 1 in 2 from 50 µM across nine wells in cell media with 1% DMSO. Control wells were made by adding DMSO to cell media to a final concentration of 1%. Cells were incubated with new media for approximately 24 h at 37℃ before analysis by flow cytometry.
After incubation with the transfection reagent, media was removed and replaced with new cell media containing the tool compound at desired concentration. Cells were incubated with the tool compound for approximately 24 h at 37℃ before analysis by flow cytometry. To prepare cells for analysis with flow cytometry, media was removed from the cells and the cells detached by incubating with 40 µL Trypsin-EDTA (0.25%, Thermo Fisher) for approximately 10 min. 50 µL of cell media was added to each well and cells were then resuspended by pipetting.

Instrument	Attune NxT (Thermo Fisher)
Software	FlowJo
Cell population abundance	No cell sorting was performed
Gating strategy	Experiments were analyzed on an Thermo Fisher Attune NxT flow cytometer. For analysis of eGFP expression, cells were gated on live singlets. To ensure only cells that were double transformed with both design-containing plasmids were included in downstream eGFP quantification, cells were then gated for the expression of mscarlett-1 and mtagBFP2. Finally, cells were gated for eGFP fluorescence.

☒ Tick this box to confirm that a figure exemplifying the gating strategy is provided in the Supplementary Information.
