# Supplementary material for: De novo design of a macrocycle-induced dimerization system for cellular control
Source: Nat Commun. 2026 May 18;17:6683. doi: 10.1038/s41467-026-71345-8 (PMC13385886; doi:10.1038/s41467-026-71345-8)
Supplement: Supplementary file 5 — Transparent Peer Review file [file 41467_2026_71345_MOESM5_ESM.pdf]

nature portfolio

Peer Review File

De novo design of a macrocycle induced dimerization system for cellular control

Corresponding Author: Professor David Baker

This manuscript has been previously reviewed at another journal. This document only contains information relating to versions considered at Nature Communications.

This file contains all reviewer reports in order by version, followed by all author rebuttals in order by version.

Version 0:

Reviewer comments:

Reviewer #4

(Remarks to the Author)
I was asked to assess the revisions for NCOMMS-25-45899-T, especially the responses to the comments of Reviewers #1 and #3, which I analysed as outlined below:

1. Reviewers 1 & 3 both raised the 'bio-orthogonality' issue (R#1-1 and R#3-1a, see excerpts of the revision summary below) and requested experimental support. I fully agree but no experiments were performed.

2. Reviewers 3 raised the 'cell permeability' issue (R#3-1b) and requested experimental support. I fully agree especially since a concentration of 50µM was needed in their cellular experiments. This is several orders of magnitude higher than existing dimerizing agents. However, no experiments were performed. This is difficult to understand since the required PAMPA or Caco-2 assay are easily obtainable as external services, as the authors have used themselves in the past.

3. Reviewer 3 asked for a better biochemical characterization (R3-2). This is crucial to assess the performance of the key compound-protein pair (MC1 & CID7) as chemical inducers of dimerization. As reviewer 3 pointed out it is especially important to determine the binary affinities of MC1 to a CID7 monomer as well as of the MC1-CID7 complex to a second CID7. This would allow to assess the cooperativity of binding, which is a key parameter for CIDs/molecular glues. The authors stated 'While we did try to further characterize the CID system in vitro in solution we were unable to determine the mechanism of the ternary complex formation.'

Unfortunately, the authors didn't state what they tried. It is hard to believe that with modern biochemical techniques the above question couldn't be answered. E.g., mass photometry should be able to distinguish between the dimeric and ternary complexes. ITC should give an indication on the stoichiometry of the formed complexes. Selective fluorescent labeling MC1 or CID7 combined with large concentration excess of one component can provide useful information. Well designed analogs to selectively abolish the ternary, but not the binary interaction could have been very powerful. In short, a number of biochemical approach could have been but weren't explored.

4. Reviewers asked for a better cellular characterization (R#1-3). The authors provide additional description of the data but no new data. It shows that the assays were run at suboptimal conditions. To really improve the manuscript it would have been necessary to repeat the reporter gene assays at lower CID7 expression levels, e.g. under the control of a weaker promotor.

5. Reviewer 3 asked to check the reversibility of the system (R#3-4). This is a key feature of CIDs. The authors relied that 'Since the readout of dimerization induction is a transcriptional response, it would be difficult to characterize the reversibility in cells as we would need to wait for GFP degradation upon ligand washout.'
This is only partially correct as the authors could have pretreated the cells with MC1 followed by washout and addition of the luciferase substrate. Moreover, it underscores that reporter gene assays are not the best assay to investigate protein-protein interaction in cells. There are many other direct techniques available such as nanoBRET, split enzyme complementation assays, Co-IP etc that could have addressed this question.

6. Reviewer 3 asked for a better description of CID field (R#1-5) and reviewer 1 asked for a more meaningful experimental comparison to existing CIDs (R#1-2). The analysis of the CID field is indeed unsatisfactory. This also is reflected in the conception of the experiments for comparison to existing CID tools. Specifically, the authors compared their MC1-CID7 system to the well established FRB-FKBP rapamycin system. However, the former is a homodimeric dimerization system, while the latter is a heterodimeric dimerization system. The FKBP12(F36V)-AP20187 system would have been the more relevant benchmark in my view, which is the gold standard for homodimerization and commercially available (e.g. https://www.takarabio.com/products/gene-function/idimerize-inducible-protein-interaction-systems/homodimerization-systems?srsltid=AfmBOoq1pay_UNVdp2HzTehSoM7Q2wAqFPm6PZ2ybN50RuBo4ArosLIE or AP20187 at MedChemExpress).
A proper analysis of the CID field incl. the FKBP12(F36V)-AP20187 system would also have shown that the 'bio-orthogonality' problem vs endogenous proteins has been solved long ago.

Taken together, while the present manuscript is another powerful demonstration what protein design can achieved nowadays, it does not surpass the existing CID tools. The biochemical and cellular characterization is superficial, clearly below the standards of Nature Communications, and not convincing to suggest its use as a CID in contemporary biological experiments (especially when compared to the established systems). Hardly any of the valid concerns of reviewer 1 and 3 were experimentally addressed.

(Remarks on code availability)

Reviewer #5

(Remarks to the Author)
Regarding the authors' response to the evaluations of reviewers 1 and 3, I would like to make the following comments.

[As Reviewer 1]
All of this reviewer's comments have been appropriately addressed. I support the publication of this manuscript.

[As Reviewer 3]
The development of the CID tool using de novo design is interesting, but experiments demonstrating the applicability of this approach in cells remain limited. Regarding concern 1, the authors should at least examine the cytotoxicity of the developed molecules and discuss the advantages and challenges compared to existing molecules. Regarding concern 4, the authors should discuss the limitations and prospects of the reversibility of the molecular assembly method developed in this study.

(Remarks on code availability)

Open Access This Peer Review File is licensed under a Creative Commons Attribution 4.0 International License, which

permits use, sharing, adaptation, distribution and reproduction in any medium or format, as long as you give appropriate credit to the original author(s) and the source, provide a link to the Creative Commons license, and indicate if changes were made.
In cases where reviewers are anonymous, credit should be given to 'Anonymous Referee' and the source.
The images or other third party material in this Peer Review File are included in the article's Creative Commons license, unless indicated otherwise in a credit line to the material. If material is not included in the article's Creative Commons license and your intended use is not permitted by statutory regulation or exceeds the permitted use, you will need to obtain permission directly from the copyright holder.
To view a copy of this license, visit https://creativecommons.org/licenses/by/4.0/

We thank the reviewers for their thoughtful feedback and comments! We addressed the concerns regarding cytotoxicity and permeability of MC1 as well as further characterized the inducibility and reversibility of the system as outlined in the point by point responses below. Our responses to reviewer comments are in blue, while the original comments from reviewers are in black.

REVIEWER COMMENTS:

Reviewer #4 (Remarks to the Author)

I was asked to assess the revisions for NCOMMS-25-45899-T, especially the responses to the comments of Reviewers #1 and #3, which I analysed as outlined below:

1. Reviewers 1 & 3 both raised the 'bio-orthogonality' issue (R#1-1 and R#3-1a, see excerpts of the revision summary below) and requested experimental support. I fully agree but no experiments were performed.

Thank you for the comment. The bio-orthogonality claim is no longer in the manuscript.

2. Reviewers 3 raised the 'cell permeability' issue (R#3-1b) and requested experimental support. I fully agree especially since a concentration of 50µM was needed in their cellular experiments. This is several orders of magnitude higher than existing dimerizing agents. However, no experiments were performed. This is difficult to understand since the required PAMPA or Caco-2 assay are easily obtainable as external services, as the authors have used themselves in the past.

We determined the apparent permeability (Pe) of MC1 to be 1.1×10^{-5} cm/s in a parallel artificial membrane permeability assay, which is above the 1.5×10^{-6} cm/s cutoff for a compound to be considered permeable (Bhardwaj G. *et al.* Accurate de novo design of membrane-traversing macrocycles. *Cell* 185, 3520–3532 (2022)). This data has been added to the supplementary text at line 120 and the Pe for MC1 and controls are plotted in Figure S2. We also reported the Pe value of MC1 at line 150 of the main text.

3. Reviewer 3 asked for a better biochemical characterization (R3-2). This is crucial to assess the performance of the key compound-protein pair (MC1 & CID7) as chemical inducers of dimerization. As reviewer 3 pointed out it is especially important to determine the binary affinities of MC1 to a CID7

monomer as well as of the MC1-CID7 complex to a second CID7. This would allow to assess the cooperativity of binding, which is a key parameter for CIDs/molecular glues. The authors stated ‘While we did try to further characterize the CID system in vitro in solution we were unable to determine the mechanism of the ternary complex formation.’

Unfortunately, the authors didn’t state what they tried. It is hard to believe that with modern biochemical techniques the above question couldn’t be answered. E.g., mass photometry should be able to distinguish between the dimeric and ternary complexes. ITC should give an indication on the stoichiometry of the formed complexes. Selective fluorescent labeling MC1 or CID7 combined with large concentration excess of one component can provide useful information. Well designed analogs to selectively abolish the ternary, but not the binary interaction could have been very powerful. In short, a number of biochemical approach could have been but weren’t explored.

We were able to measure binding affinity to the dimer via ITC, but not the monomer due to the high concentration of protein needed for the experiment. The difficulty with characterizing the assembly of the ternary complex in solution is that at concentrations required for binding measurements in experiments like FRET or ITC, the designed proteins form a homodimer.

4. Reviewers asked for a better cellular characterization (R#1-3). The authors provide additional description of the data but no new data. It shows that the assays were run at suboptimal conditions. To really improve the manuscript it would have been necessary to repeat the reporter gene assays at lower CID7 expression levels, e.g. under the control of a weaker promotor.

To address the concern of the reviewer, we used the HSV-TK promoter to control protein expression levels in a live-cell, split luciferase assay.
The HSV-TK promoter is known to induce lower levels of protein expression compared to the CMV promoter used for protein expression in the reporter assay (Promega Corporation. *NanoBiT® Protein:Protein Interaction System Technical Manual*. Technical Manual No. TM461.). We observed ~3-fold increase in luminescence signal upon addition of 2.4 μM MC1, showing CID7 is responsive to MC1. We also determined there is some background homodimerization by reading luminescence after substrate addition to cells, but before addition of MC1. Fold difference in luminescence reported is based on the change in luminescence from the signal with

and without peptide. This data has been added to Figure 3 of the main text.

5. Reviewer 3 asked to check the reversibility of the system (R#3-4). This is a key feature of CIDs. The authors relied that ‘Since the readout of dimerization induction is a transcriptional response, it would be difficult to characterize the reversibility in cells as we would need to wait for GFP degradation upon ligand washout.’
This is only partially correct as the authors could have pretreated the cells with MC1 followed by washout and addition of the luciferase substrate. Moreover, it underscores that reporter gene assays are not the best assay to investigate protein-protein interaction in cells. There are many other direct techniques available such as nanoBRET, split enzyme complementation assays, Co-IP etc that could have addressed this question.

Thank you for the comment. We were able to address the reversibility question with the Nanobit assay in HEK293T cells. As described previously, we observed ~3-fold increase in luminescence after addition of MC1 to cells pretreated with substrate. MC1 was then removed by washing the cells with 3 x 100 uL of media + 2.5% DMSO. Additional substrate was added and we observed a decrease in signal and saw the luminescent signal returned to close to the level before treatment with MC1 or vehicle. This data has been added as Figure 3 and to line 171 of the main text.

6. Reviewer 3 asked for a better description of CID field (R#1-5) and reviewer 1 asked for a more meaningful experimental comparison to existing CIDs (R#1-2). The analysis of the CID field is indeed unsatisfactory.

Thank you for providing feedback on the literature analysis. We added an additional reference to cite the AP20187 system (reference 5, main text line 33) to address this concern and strengthen the literature analysis of the manuscript. References 1-8 provide background on the usage of CIDs as well as the most commonly applied systems such as the rapamycin-FRB-FKBP, the rimiducid CID system, the AP20187 system, as well as the gibberellin and abscisic acid controlled systems from plants. References 9-13 describe the experimental efforts to engineer CID systems beyond rapamycin derivatives and the abscisic acid and gibberellin controlled CIDs. References 14-15 describe the CIDs that have been created using protein design methods.

This also is reflected in the conception of the experiments for comparison to existing CID tools. Specifically, the authors compared their MC1-CID7 system to the well established FRB-FKBP rapamycin system. However, the former is a homodimeric dimerization system, while the latter is a heterodimeric dimerization system. The FKBP12(F36V)-AP20187 system would have been the more relevant benchmark in my view, which is the gold standard for homodimerization and commercially available (e.g. https://www.takarabio.com/products/gene-function/idimerize-inducible-protein-interaction-systems/homodimerization-systems?srsltid=AfmBOoq1pay_UNVdp2HzTehSoM7Q2wAqFPm6PZ2ybN50RuBo4ArosLIE or AP20187 at MedChemExpress).
A proper analysis of the CID field incl. the FKBP12(F36V)-AP20187 system would also have shown that the 'bio-orthogonality' problem vs endogenous proteins has been solved long ago.

Thank you for the feedback. We removed the bio-orthogonality claim from the text and positioned the work described here as a proof of concept case for using protein design to generate a CID system. We do not make any claims regarding it being any more powerful than existing CID tools. We chose the rapamycin CID system as a control due to its ubiquity in the field of induced proximity. Our goal with the control experiment was to get a dynamic range of GFP fold activation for the assay.

Taken together, while the present manuscript is another powerful demonstration what protein design can achieved nowadays, it does not surpass the existing CID tools. The biochemical and cellular characterization is superficial, clearly below the standards of Nature Communications, and not convincing to suggest its use as a CID in contemporary biological experiments (especially when compared to the established systems). Hardly any of the valid concerns of reviewer 1 and 3 were experimentally addressed.
(Remarks on code availability)

Reviewer #5 (Remarks to the Author)

Regarding the authors' response to the evaluations of reviewers 1 and 3, I would like to make the following comments.

[As Reviewer 1]
All of this reviewer's comments have been appropriately addressed. I support the publication of this manuscript.

[As Reviewer 3]

The development of the CID tool using de novo design is interesting, but experiments demonstrating the applicability of this approach in cells remain limited. Regarding concern 1, the authors should at least examine the cytotoxicity of the developed molecules and discuss the advantages and challenges compared to existing molecules.

To assess the cytotoxicity of MC1, we analyzed the release of LDH from HEK293T cells treated with MC1 titrated 1 in 2 from 50 uM to 50 nM. We compared the LDH release to a vehicle only control as well as a maximum LDH control where cells were treated with TritonX 15 minutes before LDH measurements were taken. We observed no significant cytotoxicity as treatment with MC1 at all concentrations tested showed similar luminescence and percent cytotoxicity to that of vehicle-treated cells. This data has been added to the supplementary information at line 207 and the data for luminescence and percent cytotoxicity is shown in Figure S3.

Regarding concern 4, the authors should discuss the limitations and prospects of the reversibility of the molecular assembly method developed in this study.

Thank you for the comment. We were able to address the reversibility question with the Nanobit assay in HEK293T cells. As described previously, we observed ~3-fold increase in luminescence after addition of MC1 to cells pretreated with substrate. MC1 was then removed by washing the cells with 3 x 100 uL of media + 2.5% DMSO. Additional substrate was added and we observed a decrease in signal and saw the luminescent signal returned to close to the level before treatment with MC1 or vehicle. This data has been added as Figure 3 and to line 171 of the main text.
